# Gender-sensitive considerations of prehospital teamwork in critical situations

**DOI:** 10.1186/s13010-024-00153-z

**Published:** 2024-03-20

**Authors:** Matthias Zimmer, Daria Magdalena Czarniecki, Stephan Sahm

**Affiliations:** 1 Department of Internal Medicine I, Ketteler Hospital, Hesse, Germany; 2 Dr. Senckenberg Institute for History and Ethics of Medicine, Goethe-University Frankfurt, Frankfurt, Hesse Germany

**Keywords:** Patient safety, Gender, Communication, Teamwork, Error

## Abstract

**Background:**

Teamwork in emergency medical services is a very important factor in efforts to improve patient safety. The potential differences of staff gender on communication, patient safety, and teamwork were omitted. The aim of this study is to evaluate these inadequately examined areas.

**Methods:**

A descriptive and anonymous study was conducted with an online questionnaire targeting emergency physicians and paramedics. The participants were asked about teamwork, communication, patient safety and handling of errors.

**Results:**

Seven hundred fourteen prehospital professionals from all over Germany participated. A total of 65.7% of the women harmed a patient (men 72.9%), and 52.6% were ashamed when mistakes were made (men 31.7%). 19.0% of the female participants considered their communication skills to be very good, compared to 81% of the men. More women than men did not want to appear incompetent (28.4%, 15.5%) and therefore did not speak openly about mistakes. Both genders saw the character of their colleagues as a reason for poor team communication (women 89.4%, men 84.9.%). Under high stress, communication decreased (women 35.9%, men 31.0%) and expression became inaccurate (women 18.7%, men 20.1%).

**Conclusions:**

Team communication problems and teamwork in rescue services are independent of gender. Women seem to have more difficulty with open communication about mistakes because they seem to be subject to higher expectations. Work organization should be adapted to women’s needs to enable more effective error management. We conclude that it is necessary to promote a positive error and communication culture to increase patient safety.

**Supplementary Information:**

The online version contains supplementary material available at 10.1186/s13010-024-00153-z.

## Introduction

Teamwork is the central pillar of patient care in emergency medical services (EMS). As in other high-risk workplaces, the quality of teamwork depends significantly on communication among team members. However, communication, teamwork, and ways of addressing errors in the context of EMS have received insufficient study from the perspective of gender.

The aim of the study is to describe experiences with errors, teamwork and communication on German EMS teams from the perspective of gender. Hitherto, very little usable data regarding this important area of the healthcare system have been made available.

Nevertheless, such an approach risks bias due to traditional stereotypes concerning female and male communication. The performance expectations associated with typical female communication behavior shape our decisions to a greater extent than do women’s objective performance [1]. Stereotypes are socially shared beliefs regarding the characteristics of a group. The group membership of the individual, which is associated with certain characteristic features, outweighs the features of the individual who belongs to such a group (e.g., the characteristics of women outweigh the characteristics of female paramedics) [2]. Men are described as assertive, competitive and independent, whereas women are described as warm, friendly and supportive [3]. Accordingly, the presence of these characteristics could lead to differences in communication, team behaviors and ways of addressing errors. However, no concrete research has investigated this topic. Experiences with mixed gender team communication from high-risk nonmedical workplaces should not be transferred to the health care sector without reflection [4].

Women did not attain a noticeable presence in German EMS until the first decade of the 21st century. As in fire department or police services, women were not employed in this field or were employed only in highly specialized roles [5]. This horizontal segregation has changed only slowly over time. In his static description of EMS in Germany in the year 2000, Behrendt lists no female paramedics [6]. The only reference to women in EMS is the summary designation of medical staff as male/female emergency physicians [6]. A continually advancing social change in this context [7] is that the number of female employees is increasing. Comparable changes have also been observed internationally, for example, in the USA [8]. At present, 30.9% of paramedics in Germany are female, and 62.4% of medical students are already women [9].

## Methods

This study employed a descriptive, anonymous and voluntary design. To conduct empirical social research, we used a standardized, nonvalidated questionnaire survey to ensure a high degree of objectivity in the implementation of this research. The target group included paramedics and pre-hospital emergency physicians. The participants were asked about their experiences with their own behaviors and the behaviors of others with which they had personal experience in the EMS context. The individual test items were not operationalized to represent specific characteristics. Participants agreed that their data could be used for scientific research. The data were stored directly on a server located at a SOC 2-accredited data center in a completely anonymized manner. The data center was ISO 27001 certified and was subject to the General Data Protection Regulation (GDPR). The collected data set could not be traced back to single individuals. Participants received no financial benefits in return for their participation. The study was conducted in accordance with the principles of the Declaration of Helsinki. The Ethics Committee of the State Medical Association of Hesse did not consider a formal ethical review of the study to be necessary, as the data were collected anonymously (decision reference number FF67/2016).

The development of the questionnaire was based on 17 assumptions regarding communication and patient safety in the EMS context that were formulated by the authors. The individual items included in the questionnaire were based on a detailed literature review that focused on the main areas of communication and risk management. The questions were divided into the main categories of consent to the study (one question), the sociobiological data of the participants (5 questions), rescue service structures (2 questions), communication (19 questions), teamwork (7 questions), error management (9 questions) and training (10 questions). The main category of communication included the subcategories of team communication (16 questions) and handover (3 questions). The analyzed questions analyzed as part of this research were thematically related to attitudes regarding experiences with errors while on duty in EMS, harm to patients during care, communication within the team, and the causes of communication deficits. Not all the questions were included in the evaluation discussed in this article, as they were not all related to the topic under discussion. The questionnaire included 3 open-ended, 38 single-choice and 12 multiple-choice questions. The focus of the questionnaire was on the subjective impressions of the participants. In the questions concerning the length of injury, we provided brief examples to make it easier for participants to classify their subjective impressions. The test structure was subjected to an expert review. As members of Goethe University Frankfurt, we consulted on consultation on the development of the questionnaire with the Institute for Biostatistics and Mathematical Modeling at the Center for Health Sciences of Goethe University Frankfurt.

We sent nonpersonalized invitations by mail to the management of German EMS stations throughout Germany as well as to regional EMS medical directors asking them to forward the invitations to their staff. The invitation letters contained an internet link to the online questionnaire.

### Inclusion criteria

1. Paramedics (PMs)
2. Prehospital emergency physicians (EPs).
3. Employees of a German ambulance service.
4. Voluntary and unpaid participation in the study.
5. Agreement with the data protection regulations.

### Exclusion criteria

1. Failure to meet at least one of the inclusion criteria.

### Data analysis

In light of the descriptive, noninterventional nature of the study, no hypothesis-based case number calculation was performed. The categorical characteristics of the two groups were examined using the chi-square test and the Fisher-Freeman-Halton exact test. For nonparametric variables, a Spearman correlation analysis was performed. All the statistical tests were two-sided and performed at a significance level of 0.05. The data were organized using Microsoft® Excel software 2016 for Windows (Microsoft Corporation, Redmond, USA). BiAS version 11.06 software (epsilon-Verlag, Frankfurt, Germany) was used to conduct the statistical analyses.

## Results

A total of 722 questionnaires were returned. The participants returned 8 questionnaires that were empty. The remaining 714 questionnaires met the inclusion criteria and were included in the analysis. The completion rate was 0.98.

Among the participants, 17.9% (128) were women and 82.1% (586) were men (*p* = 0.01). The participants had a mean age of 35.9 ± 10.5 years and a mean work experience of 12.5 ± 9.4 years (Table 1). Participants perceived their work in EMS to be demanding (female 96.8%, 124; male 90.5%, 530; *p* = 0.01).

**Table 1:** Participants

	**Female**	**Male**
Age, years	33.2 ± 10.1	36.3 ± 10.4
Professional experience, years	8.7 ± 7.6	13.2 ± 9.5
PM	16.0%	84.0%
EP	23.0%	77.0%

### Patient harm

When asked about their own experiences with and fear of patient harm, gender differences among the participants emerged (Table 2).

**Table 2:** Harm to patients and fear of consequences of harm

	**Female**	**Male**	***P*-value**
“Have you ever caused harm to a patient?”			
No	34.3% (44)	27.1% (159)	0.09
Yes, harm unknown	15.6% (20)	16.8% (98)	0.76
Yes, short-term harm	46.8% (60)	57.6% (338)	0.02
Yes, medium-term harm	5.4% (7)	6.9% (40)	0.57
Yes, long-term harm	4.6% (6)	5.8% (34)	0.61
“How great are your fears of legal, employment, or civil sanctions if a patient were harmed as a result of your work?”			
Great fear of short-term harm	78.9% (101)	85.1% (499)	0.08
Great fear of medium-term harm	42.9% (55)	50.5% (296)	0.12
Great fear of long-term harm	20.3% (26)	23.5% (138)	0.43
Why wouldn’t you be open about mistakes?			
Fear of legal sanctions	48.4% (62)	44.9% (263)	0.46
Fear of employment law sanctions	42.1% (54)	40.8% (239)	0.77
Shame in front of colleagues and superiors	52.6% (67)	31.7% (186)	< 0.01
No appreciation from colleagues for admission of mistake	44.2% (57)	37.5% (220)	0.14
Errors or similar are not an issue for me	5.3% (7)	5.8% (34)	0.88
Shame and the subject do not interest me	3.2% (4)	15.2% (89)	< 0.01

### Communication error

Men and women were equally interested in the topic of communication (female 70.4%, 90, male 79.8%, 468; *p* = 0.01). In this context, 19.0% (24) of the female participants perceived their own communication skills to be very good, whereas 81.0% (475) of the men expressed such a perception (*p* < 0.01). Women seem to be more concerned than men about maintaining their competence, shame or fear of sanctions with regard to their own communication (Table 3).

**Table 3:** Deficits of own communication and how to deal with them. Multiple responses possible

	**Female**	**Male**	***P*-value**
If you forgot something you were told once in patient care, why didn’t you ask again?			
“There wasn’t time.”	34.7% (44)	35.3% (207)	0.83
“There are too many tasks I have to handle at the same time.”	62.1% (79)	57.8% (339)	0.42
“I don’t want to seem unfocused.”	16.8% (22)	14.1% (83)	0.38
“I don’t want to seem incompetent.”	28.4% (36)	15.5% (91)	< 0.01
“I think inquiring is unnecessary.”	3.2% (4)	5.2% (30)	0.33
“I’ve never forgotten anything.”	9.5% (12)	14.3% (84)	0.13
Imagine you misspoke in patient care and there was harm to the patient because of it (e.g., you requested amiodarone when you meant epinephrine). How do you feel afterwards?			
“I feel bad because well-trained staff shouldn’t make mistakes.”	85.7% (110)	84.9% (498)	0.78
“I don’t think it’s a bad thing because mistakes are part of everyday life.”	8.4% (11)	12.0% (70)	0.27
“I don’t want to look bad in front of colleagues.”	18.5% (24)	10.9% (64)	0.01
“I feel ashamed”	47.1% (60)	35.5% (208)	0.01
“I am afraid of sanctions”	42.9% (55)	28.7% (168)	< 0.01

In the context of patient handover, 43.1% (55) of female participants felt that others listened them to less than their male colleagues. This rate was as high as 50.0% for nonphysician participants and as low as 31.1% for female emergency physicians. With slight increasing age (female rho 0.30, men rho 0.23) and service experience (female rho 0.22, men rho 0.17), female participants were less likely to report interruptions to patient handoff. Gender differences were observed, particularly with regard to experiences with the communication behavior of other team members (Table 4).

**Table 4:** Experienced and own communication behavior

	**Female**	**Male**	***P*-value**
Experienced communication behavior.			
Asking for feedback from team members after a difficult patient care.	85.9% (110)	82.0% (481)	0.29
Hear statements from colleagues that are not appreciative.	9.3% (12)	4.9% (29)	0.05
Experiencing increased interruption in patient care and handoffs.	33.5% (43)	40.2% (236)	0.16
Own communication behavior.			
Repeating a received task before performing it.	36.7% (47)	44.3% (260)	0.11
Loud announcement when an assigned task has been completed.	73.4% (94)	72.8% (427)	0.89
Addressing colleagues by name when a task is passed on.	83.5% (107)	79.6% (466)	0.29
Forgetting communicated information during patient handoffs.	12.5% (16)	13.4% (79)	0.76
Forget to hand over information at the time of delivery.	12.5% (16)	12.9% (76)	0.88
Twisting of information during the handover.	0.7% (1)	2.5% (15)	0.32
Confusion under high stress	5.4% (7)	5.9% (35)	0.82
Wrong listening under high stress	7.0% (9)	10.2% (60)	0.26
Inaccurate expression high stress	18.7% (24)	20.1% (118)	0.72
Decreased communication under high stress	35.9% (46)	31.0% (182)	0.28
Take a wrong tone under high stress	10.1% (13)	9.0% (53)	0.69
Mistakes, slips or errors under high stress	10.1% (13)	8.3% (49)	0.51

Overall, 60.9% (76) of the female and 55.4% (325) of the male participants considered the EPs to be good team players (*p* = 0.41). This finding was based on the participants’ own subjective impressions. In contrast, 92.6% (119) of females and 91.8% (538) of males considered PMs to be good team players (*p* = 0.66). Finally, 11.7% (15) of female participants (male 9.8%, 57; *p* = 0.49) indicated that certain team constellations led to more frequent communication errors (Table 5).

**Table 5:** Which of these causes that can lead to poor professional communication have you personally faced?

	**Female**	**Male**	***P*-value**
Leadership behavior	56.9% (73)	68.2% (400)	0.01
Work and business organization	38.2% (49)	54.0% (316)	< 0.01
Character of colleagues	89.4% (114)	84.9% (498)	0.23
Lack of training opportunities on this topic	26.8% (34)	36.0% (211)	0.04
There is too little awareness of training on this topic	17.9% (23)	26.1% (153)	0.05
Character of own person	39.8% (51)	33.5% (196)	0.16

## Discussion

The ratio of female to male participants in the study reflects the gender distribution in the German EMS accurately [9]. Regardless of gender, the participants perceived their work in EMS to be demanding and expressed a high level of interest in the topic of communication. Therefore, participants of both genders can be assumed to exhibit identical professional self-images, as described by Koch et al. [10]. Women and men complete the same training and work in the same work environment. Therefore, it can be concluded that the observed differences in perceptions and self-assessments could be based on gender.

### Patient harm

Recalled experiences of patient harm were distributed similarly between men and women. Individuals of both genders were more likely to experience short-term harm, and men were significantly more likely to do so. Whether the women included in our study are somewhat more careful with regard to their patients, as Tsugawa et al. or Rouse et al. [11, 12] indicated, or believe that they are more careful (e.g., to conform to societal expectations) cannot be clarified at present. The women who participated in this research seem to be more likely than the men to be influenced by the shame associated with causing harm and their fear of their own incompetence. According to fuzzy-trace theory, this situation could also influence their memories [13]. This theory suggests that memories are not free from manipulation or deviation in terms of either their core meaning or their specific details [13, 14]. Accordingly, it is possible that women actually do cause less harm. However, because of shame or fear, they believe that they are causing more harm.

To address patient harm, women seem to place more emphasis on an open approach to errors. It is possible that women are more accepting of an open error culture, which may improve team performance in the long term [15]. No gender-specific differences in the fear of sanctions pertaining to different degrees of patient harm were observed. Curiously, fear decreased with the severity of patient harm regardless of gender. This finding could be due to a lack of experience with such severe harm on the part of participants. No gender-dependent risk perceptions were observed.

### Error management

Regardless of gender, participants had high expectations of their own professionalism. As a result, they may perceive errors as eliciting an emotional burden. Such emotional burden could make open communication difficult. This situation generates psychological insecurity that impedes all efforts to improve safety in the workplace [16].

Equal numbers of women and men were generally uninterested in addressing errors openly. Men reported that they were ashamed of errors significantly more often and were therefore uninterested in addressing errors through open discussions. One possible explanation for this finding could be male role conflict (between the role of the perfect man who exhibits zero tolerance for errors vs. that of the professional paramedic who engages in open communication), although the available data cannot clarify this issue.

Women seem to be more strongly impacted by their desire to avoid appearing incompetent. Of course, we must remember that women must assert themselves in a male-dominated environment. It is conceivable that women could experience stronger feelings of supposed incompetence and shame due to their position as a minority in EMS contexts [17].

Regardless of employees’ gender, an open error culture should be promoted intensively in EMS contexts. If feelings of supposed incompetence and shame interfere with the factual processing of errors, such causal obstacles must be removed. Adverse events, mistakes, etc., should be discussed openly to ensure that their consequences can be observed [18].

Making an error in a high-risk workplace is not necessarily a sign of incompetence. However, concealing an error gives the appearance of incompetence.

With regard to female employees and their self-assessment, a continued increase in their presence in the workforce could be helpful. Such an increase could reduce their minority status and thus improve their self-assessments [17].

Furthermore, operational conditions should be adapted to the needs of female employees [12]. For example, supervisors could focus more on topics such as shame due to errors and perceived incompetence in employee appraisals. Employees could be guided to recognize their feelings in an appropriate manner. In training, paramedics should be taught that gender does not determines competence or the occurrence of errors; rather, our nature as human beings does so. All employees working in EMS should learn to live in an open error culture, admit to the errors to which they have contributed and proactively recognize the beginnings of errors in the workplace [18].

### Communication

Interest in the topic of communication is equally high regardless of gender. Men identify themselves as having better communication skills. This differences could be due to the stereotypical gender-associated behavioral spectrum [13, 18], which both men and women use as a reference. The specifics of this behavioral spectrum are ingrained in the collective consciousness of society, reinforced by popular scientific traditions and based in part on scientific findings reported during the second half of the 20th century [19–21]. The performance expectations of typical female communication behavior shapes our decisions more than does women’s objective performance [1]. However, the conclusions of those studies could have been influenced by the unequal social positions of women and men at that time. In general, women’s performance is underestimated on a gender-specific basis if their performance is not truly addressed objectively [22]. In addition, Bergmann et al. conducted simulation studies that revealed that considerable cognitive effort is required to separate the effects of female gender from the effects of doubts about competence, a connection which is inherent in large portions of the population [23]. To solve this dilemma, we recommend consistent training in the ability to reflect on both one’s own communication and that of others. Modern findings from communication research should be incorporated into such training, and old thought patterns must be overcome in the long term.

Regardless of gender, the main factor associated with poor communication was task plurality in the context of patient care and handoff. While female participants were more likely to complain about inattention on the part of their interlocutors, no significant differences between the genders were observed in terms of complaints about interruptions during patient handover. Koch et al. were able to show explicitly that these self-perceptions can be deceptive with respect to interruptions of one’s own flow of speech and the distribution of the nonverbal attention of one’s dialog partner [10]. However, despite these findings, we believe that for targeted countermeasures can be developed based on the significant number of reports of disruptions in conversation.

Standardized and focused communication and coordinated work could lead to reductions in cognitive load adverse events. In line with the sterile cockpit rules used in aviation, rules of conduct for EMS teams in the field should be developed [24]. In aviation, these rules require a focus on the control and implementation of actions in complex situations (e.g., during take-off or landing). Both genders should communicate explicitly and use precise wording, I-messages, and closed feedback loops. In addition, we recommend that relevant actors should pay active attention to the interlocutor, let the interlocutor speak and ensure congruence in terms of verbal and nonverbal attention.

A harmonized language code for patient handoff should be established regardless of gender. This approach could help neutralize the disruptive character traits of participants in patient handover as well as asymmetric communication relationships (PMs vs. EPs, PMs vs. nurse, prehospital EPs vs. emergency room physician). The soft skills needed for this purpose must become an integral part of professional training for all professions.

### Team communication

Women experience less appreciative communication. Unfortunately, our study cannot determine the direction from which such less appreciative communication comes (e.g., team members, the emergency department, patients, or relatives). One team-based cause of such communication might be the fact that women represent a minority in EMS. This minority position can be a source of lower status for women on teams and can lead to less appreciative communication. Heads of rescue services and heads of emergency rooms should generally pay attention to respectful communication within their teams. However, patients and teamwork benefit from mixed-gender teams [15]. According to the theory of critical mass, even more women should enter professions in the field of EMS to facilitate culture change [25].

The approach of closed loop communication [26] was used equally by both genders. Overall, this situation continues to exhibit the potential for improvement with regard to enhancing the higher degree of use of this style of communication. Information was also forgotten regardless of gender. Although men seem to be more likely to distort information during patient handoff, the number of participants affected is in the low single digits. The same is true with regard to the influence of stress on the quantity and quality of communication. All of these findings indicate that women and men employ similar information processing and are equally dependent on environmental factors. In particular, reduced communication under conditions of stress and imprecise formulations are serious threats to patient safety. Therefore, we do not view gender-specific educational measures as necessary but rather focus on the general development of positive coping strategies for stressful situations. In addition, crew resource management should be implemented to support the communication skills of both male and female emergency service personnel. We recommend targeted training in team communication regardless of gender.

### Teamwork

A slight majority of participants view emergency physicians as good team players, and no differences were observed between genders in this context. In comparison, among both men and women, paramedics are viewed as good team players by more than 90.0% of the respondents.

Both genders attribute poor teamwork to similar causes. However, men report slightly more problems with leadership, work organization and training opportunities. Nevertheless, this finding confirms the existence of a relatively gender-independent presumption of causes. Thus, preclinical results contrast slightly with findings drawn from the clinical context o concerning n female residents, who must struggle disproportionately to obtain recognition within their teams [27]. When selecting employees in the EMS context, character traits should be emphasized more strongly. Especially with regard to the selection of emergency physicians, who automatically become team leaders in Germany, character traits pertaining to leadership should be considered. In addition, existing work and organizational structures must be examined to detect both positive and negative influences on communication. Participants, on the other hand, are less likely to believe that insufficient knowledge about good communication. In principle, the German training curriculum for paramedics [28] includes learning units on team communication, while the curriculum for physicians does not. Regardless of gender, the participants associated such problems more closely with the daily implementation of the learned knowledge. Thus, each EMS manager and individual employee must ensure that the apparently well-known rules for good teamwork are applied in daily work.

Communication problems in EMS are largely independent of the gender of employees. Such differences are more likely to exist with regard to employees’ perceptions of their own inadequacies. For women, addressing errors in patient care and failed communication seem to be associated with greater obstacles. Open communication concerning one’s own errors is more difficult for women than for men. From the participants’ perspective, the personalities of team members are also crucial for good teamwork. Gender is not an important criterion for good teamwork. Baker came to a similar conclusion in the military environment [29].

We conclude that establishing a positive error and communication culture is necessary to promote better teamwork. The organization of work could be adapted slightly more to the needs of women. Two important reasons for this claim are that women are more ashamed of communication deficits and more afraid of sanctions than men. If shame and fear hinder the flow of information within a team, then communication-based teamwork could deteriorate. Possible adjustments could include holding discussions about mistakes in a protected environment, addressing fears in a more sensitive manner during discussions and offering protection against sanctions. These approaches could be accompanied by repeated training on the topics of error management, team communication and discussion culture.

## Limitations

Due to the study design, it should noted that more ambulance staff who were interested in the topic of communication may have participated in this research. Employees with less motivation or disinterest in communication may have refused to participate in the study.

Moreover, participants’ self-judgments are subject to a halo effect, which refers to an apparent perception of themselves [30]. Thus, categorizing of one’s own gender can generate a false intrapsychic judgment process in cases featuring is sufficient correspondence with perceived stimuli. False memories can be generated that distort can self-perceptions [28].

The study did not examine the different linguistic skills of EMS staff, such as expression, vocabulary, and language comprehension, which have an impact on professional communication.

## Conclusion

Patient harm, communication problems and teamwork in EMS contexts are largely independent of employees’ gender. Differences are more likely to emerge employees’ perceptions of their own inadequacies. For women, addressing errors in patient care and failed communication seem to be associated with greater obstacles. Open communication about one’s own errors is more difficult for women than for men. On the other hand, men sometimes do not want to address mistakes because the subject causes them to experience shame. From the participants’ perspective, the personalities of team members are also crucial for good teamwork. Gender is not an important criterion for good teamwork. We conclude that stimulating a positive error and communication culture without shame or fear regarding the possibility of conveying an impression of poor professionalism is necessary in response to patient harm. A further increase in the proportion of women in EMS would be helpful with regard to resolving the psychological effects of the minority status of women. All employees working in the EMS context should learn that gender is not the decisive factor with regard to their ability to work on a team; rather, their mindset is key.

### Supplementary Information

**Supplementary Material 1.**

## Data Availability

All data generated or analysed during this study are included in this published article and its supplementary information files.

## Abbreviations

EMS: Emergency medical services
EP: Prehospital emergency physicians
PM: Paramedic
